# Emotional Infant Face Processing in Women With Major Depression and Expecting Parents With Depressive Symptoms

**DOI:** 10.3389/fpsyg.2021.657269

**Published:** 2021-07-02

**Authors:** Agnes Bohne, Dag Nordahl, Åsne A. W. Lindahl, Pål Ulvenes, Catharina E. A. Wang, Gerit Pfuhl

**Affiliations:** ^1^Department of Psychology, Faculty of Health Sciences, UiT the Arctic University of Norway, Tromsø, Norway; ^2^Modum Bad Research Institute, Vikersund, Norway

**Keywords:** attentional bias, mood-congruent, recognition memory, baby schema, prenatal depression, rumination

## Abstract

Processing of emotional facial expressions is of great importance in interpersonal relationships. Aberrant engagement with facial expressions, particularly an engagement with sad faces, loss of engagement with happy faces, and enhanced memory of sadness has been found in depression. Since most studies used adult faces, we here examined if such biases also occur in processing of infant faces in those with depression or depressive symptoms. In study 1, we recruited 25 inpatient women with major depression and 25 matched controls. In study 2, we extracted a sample of expecting parents from the NorBaby study, where 29 reported elevated levels of depressive symptoms, and 29 were matched controls. In both studies, we assessed attentional bias with a dot-probe task using happy, sad and neutral infant faces, and facial memory bias with a recognition task using happy, sad, angry, afraid, surprised, disgusted and neutral infant and adult faces. Participants also completed the Ruminative Responses Scale and Becks Depression Inventory-II. In study 1, we found no group difference in either attention to or memory accuracy for emotional infant faces. Neither attention nor recognition was associated with rumination. In study 2, we found that the group with depressive symptoms disengaged more slowly than healthy controls from sad infant faces, and this was related to rumination. The results place emphasis on the importance of emotional self-relevant material when examining cognitive processing in depression. Together, these studies demonstrate that a mood-congruent attentional bias to infant faces is present in expecting parents with depressive symptoms, but not in inpatients with Major Depression Disorder who do not have younger children.

## Introduction

Human survival depends on infants drawing attention from adults. Infants' cues are of high evolutionary significance and processing of infant faces is assumed to be particularly prominent. Lorenz (1943) termed this innate mechanism the “Kindchenschema” or baby schema. This mechanism attracts attention and care behaviors toward infants (Lorenz, 1943; Luo et al., 2011). Infant faces also take attentional precedence compared to adult faces (Brosch et al., 2007; Thompson-Booth et al., 2014b) and compared to faces of older children (Thompson-Booth et al., 2014a). The baby schema is present in both parents and non-parents (Kringelbach et al., 2008; Lucion et al., 2017), although some studies find a stronger effect in parents (Thompson-Booth et al., 2014a,b). Brain areas associated with face processing and attention respond more strongly to infant faces than adult faces (Leibenluft et al., 2004; Kringelbach et al., 2008; Luo et al., 2015). In summary, infant faces are considered highly salient stimuli, and this finding is consistent across different tasks and designs (for a review, see Lucion et al., 2017). Less is known about how mental illness, in this case depression, might alter the cognitive processing of infant faces. Maternal depression is associated with a range of adverse child outcomes (Goodman et al., 2011), and Stein et al. (2009) argue that this transmission of risk might be caused by maternal cognitions that characterizes depression. One possibility is that the baby schema ensures normal infant face processing in depression.

### Processing of Infant Faces in Depression

Individuals with major depression tend to process adult faces mood-congruently, that is they demonstrate a preferential processing of negative material (Gotlib and Joormann, 2010; LeMoult and Gotlib, 2019). This mood-congruent bias affects attention to, recognition memory of, and interpretation of adult emotional faces (Bistricky et al., 2011). Only a few studies have investigated infant face processing in individuals with depression or depressive symptoms (for a review, see: Webb and Ayers, 2014). To the best of our knowledge no study has investigated bias in recognition memory of infant emotional faces. Most studies to date examined interpretation of infants' emotional expressions and found differences between depressed and non-depressed women. Arteche et al. (2011), using a morphed infant face task, found that mothers with postnatal depression were less accurate in identifying happy infant faces than the control group. However, there were no difference in accuracy to sad faces. Broth et al. (2004) found that higher levels of self-reported depressive symptoms in clinically depressed mothers were related to less accuracy in identifying positive infant emotional expressions. Stein et al. (2010) found that mothers diagnosed with depression rated negative infant facial expressions as more negative than controls in a long duration presentation (2,000 ms), but not when using a short presentation (100 ms). Together, these studies imply a mood-congruent interpretation bias of infant faces in depression, in line with research on adult faces.

Attentional bias toward a stimulus indicates that the stimulus causes greater cognitive engagement, in that it captures or holds the attention to a greater extent than other stimuli. Depressed individuals seem to be more engaged with stimuli that confirm their state, especially mood-congruent material (Gotlib and Joormann, 2010; LeMoult and Gotlib, 2019). However, the baby schema would make another prediction, namely similar or greater engagement with distressed compared to neutral infant faces in healthy adults. Indeed, non-depressed pregnant women disengaged more slowly from distressed infant faces, than happy or neutral ones in a go/no-go attentional task (Pearson et al., 2010). Women with depressive symptoms did not show the same bias. Pearson et al. (2013) repeated their finding in a group of (pregnant) women diagnosed with Major Depression Disorder (MDD). In this study, they also intervened by treating half the depressed group with cognitive behavioral therapy (CBT). The CBT-group displayed the same attentional bias toward distressed infant faces as the control group post-intervention.

Of note, in the study by Pearson et al. (2010, 2013) a stimulus presentation of 240 ms was used, but for a mood-congruent attentional bias in processing of adult faces to emerge, a stimulus presentation of at least 1,000 ms is required (Bradley et al., 1997; Mathews and MacLeod, 2005). In fact, there is evidence that depressed individuals are not characterized by biases in early or subliminal processing (Gotlib and Joormann, 2010). Accordingly, Dudek and Haley (2020) failed to replicate the results of Pearson et al. applying the same go/no-go attentional task with the 240 ms presentation time. They found that neither reaction time nor ERP-responses to infant faces were related to depression and anxiety scores, respectively. However, in a study presenting mothers with images of both their own and other infants, Laurent and Ablow (2013) found reduced neural activity to both infant joy and distress in mothers with depressive symptoms. Stimulus presentation in this study was 6 s. This could indicate less engagement with faces in general, or flat affect among the depressed women, which has been recognized as a risk factor in parent-infant interaction in maternal depression (Feldman, 2007).

Thus, depressed individuals show a mood-congruent attentional bias for adult faces, while data for infant faces is sparse and inconsistent. The inconsistency might be due to using inappropriate stimulus presentation times and using non-standardized infant face images. As the interpretation biases to infant faces in depression seem to be the same as to adult faces, we think it likely that the attentional bias is also mood-congruent with sufficient stimulus presentation time. The baby schema might reduce the mood-congruent attentional bias but based on the above we still expect depression to affect the processing. Mood-congruent biases are also related to rumination, which we review below.

### Processing Biases and Rumination

Nolen-Hoeksema et al. (2008) argued that mood-congruent cognitive biases in depressed individuals could be associated with depressive rumination, as both involve being caught up in negative associations. Rumination refers to the tendency to repetitively focus on distress and the possible causes and consequences of it, instead of active problem solving, and this thinking style is strongly and consistently related to depressive symptoms (Nolen-Hoeksema et al., 2008). Koster et al. (2011) proposed “the impaired disengagement hypothesis.” This hypothesis explains both rumination and cognitive biases in depression as a result of lower attentional control in the presence of self-relevant negative information, and therefore difficulties disengaging from such material. Joormann (2010) explains rumination, biases to negative material and emotional regulation difficulties as an effect of poor cognitive inhibition. Accordingly, studies have found an association between depressive rumination and a mood-congruent attentional bias to words (Donaldson et al., 2007; Grafton et al., 2016) and adult faces (Joormann et al., 2006). Grafton et al. (2016) demonstrated that this attentional bias indeed was an impaired disengagement as opposed to facilitated initial engagement with negative material. This is also found in a review of eye tracking studies (Suslow et al., 2020). However, one study failed to show an association between rumination and mood-congruent attentional bias (Goeleven et al., 2006). The authors suggest that this could be explained by the different nature of the measures. The attention task uses visual stimuli, here faces, whereas rumination is a more verbal process. The link between attentional bias and rumination remains unclear. To the best of our knowledge, no studies investigated if and how depressive rumination and bias in recognition memory are interrelated.

### The Present Studies

There is a substantial lack of knowledge on how depression or depressive symptoms affect the processing of infant faces. In two studies presented below we aim to shed light on facial processing of infant faces in depression. In the first study, we investigated biases in attention and recognition of emotional infant faces in a group of inpatients with an MDD diagnosis and a group of healthy controls. In the second study, we wanted to test the same hypotheses in a group of expecting parents with and without depressive symptoms, as infant faces should be of higher relevance to them than the patient group in study 1.

To examine attentional bias, we used a version of the emotional dot probe-task, commonly applied in measuring attentional bias in both anxious and depressed individuals (Mathews and MacLeod, 2005). However, the reliability and validity of the task is debated, with low reliability at the individual level reported for studies applying the dot-probe task in anxious individuals (Schmukle, 2005; Staugaard, 2009; Waechter et al., 2014; Price et al., 2015). Even so, these studies concluded that the dot-probe task still might be acceptable for investigation of differences at the group-level (Schmukle, 2005; Staugaard, 2009; Price et al., 2015). Importantly, there are essential differences in the attentional biases observed in anxious and depressed individuals (Mathews and MacLeod, 2005). Anxiety is related to attentional bias in initial attention to angry (threatening) or fearful faces and can be observed with short stimulus presentation times. Depression on the other hand is related to attentional bias only to sad faces and is only observed with longer stimulus presentation time. The present studies investigated attentional bias to emotional infant faces with sufficient stimulus presentation (1,000 ms; Mathews and MacLeod, 2005; Gotlib and Joormann, 2010) and no competing stimulus (see Method section) to directly measure disengagement.

To examine recognition of emotional infant faces, we used a version of the Deese-Roediger-McDermott (DRM) paradigm (Deese, 1959; Roediger and McDermott, 1995), which previously has been shown to yield both mood-congruent free recall (Joormann et al., 2009) and recognition (Howe and Malone, 2011) with word lists as stimuli. In their study with word lists, Howe and Malone (2011) found that participants with depression remembered words from negative and depression-related lists better than words from neutral or positive lists. They also falsely remembered more depression-related words not being part of the list. The recognition memory of the non-depressed control group was unaffected by list valence. In our recognition task we used a similar set up as Howe and Malone (2011), but used emotional adult and infant faces instead of words.

Studies on attentional bias to adult faces and infant faces are in conflict, and we believe this might be because of the stimulus presentation time was too short in the studies on infant faces (Pearson et al., 2010, 2013; Dudek and Haley, 2020). Research on interpretation bias seems to largely find the same mood-congruent bias in depression, regardless of the stimuli being adult or infant faces. No studies have examined bias in recognition memory of infant faces. Our predictions are therefore in line with the knowledge of biases to adult faces in depression. Specifically, we hypothesized that a mood-congruent attentional bias would cause women with MDD (study 1) or the depressed group (study 2) to (1) disengage faster than healthy controls from happy infant faces, and (2) disengage more slowly than healthy controls from neutral and sad infant faces. Further, a mood-congruent memory bias would cause persons with depression to (3) recognize infant faces expressing a negative emotion more accurately than healthy controls, and (4) recognize infant faces expressing positive emotions less accurately than healthy controls. Finally, we hypothesized that (5) greater levels of rumination would be related to more severe levels of depression, and (6) rumination and depression severity would be related to biases in attention and memory.

Previous studies found significant group differences for attentional bias in the dot probe task with 23–26 patients and 19–25 matched controls (Gotlib et al., 2004; Joormann and Gotlib, 2007; Ao et al., 2020) We therefore aimed to recruit at least *n* = 25 participants in each group for both studies.

## Study 1: Biases in Major Depressive Disorder

The purpose of study 1 was to examine if women with MDD demonstrate attentional and memory biases when processing emotional infant faces, and if so, if the biases are mood congruent and related to rumination.

### Materials and Methods

#### Participants

Two groups of participants were recruited: a depressed group consisting of 26 women diagnosed with MDD, and a control group of 25 healthy women. One patient abandoned testing after the first task and was excluded. The depressed group was recruited from an inpatient depression-treatment facility. The facility had few male patients. We therefore only included female participants to ensure anonymity in compliance with GDPR regulations. The patients in this facility all fulfill ICD-10 criteria for MDD, recurrent; and dysthymia could be comorbid with this disorder. These patients are non-responders to earlier in- or outpatient treatments, and many had previous unsuccessful attempts with pharmacotherapy. As part of the facility's routine practice, patients were instructed to discontinue benzodiazepine and other addictive medication before the start of psychotherapy. Exclusion criteria were current suicidal risk, current psychosis, marked emotional instability (e.g., issues with impulsivity), strong paranoid traits, and current problems related to heavy substance abuse. Symptoms of other psychiatric disorders, for instance anxiety, have not been controlled for as this complicated the recruitment of a clinical sample. However, all participants in the depressed group had MDD as their primary diagnosis. Patients were between 18 and 65 years old.

The healthy control group, also all women, was recruited through social media, the university web page and snowballing. The healthy group was recruited after the patient group and matched to the patient group in age and parenthood. In the patient group, 15 participants had children, one woman had a child under the age of 3. In the control group, 15 participants were parents, and there was one woman who had a child under the age of 3. For participant characteristics, see Table 1.

**Table 1:** Participant characteristics study 1.

	**MDD group**	**Control group**
	***N***	***N***
**Age**		
18–30	8	8
31–40	3	3
41–50	6	6
51+	8	8
Higher education^a^	13	20
	***M (SD)***	***M (SD)***
BDI-II	24.8 (9.4)	5.1 (4.4)
RRS	56.1 (12.4)	34.2 (9.1)

a *Completed 3 years or more in university or college*.

#### Stimuli

Photographs of faces from infants aged 4–12 months old were taken from the Tromsø Infant Faces database (TIF; Maack et al., 2017). TIF consists of standardized images of infants displaying a range of emotional facial expressions, including sadness, happiness, fear, anger, surprise, disgust, and neutral. These images were used as stimuli in both the attentional and the recognition task. The TIF database is comparable to databases using adult emotional facial expressions like the Karolinska Directed Emotional Faces (KDEF; Lundqvist et al., 1998).

#### Dot-Probe Task

**Figure 1:**
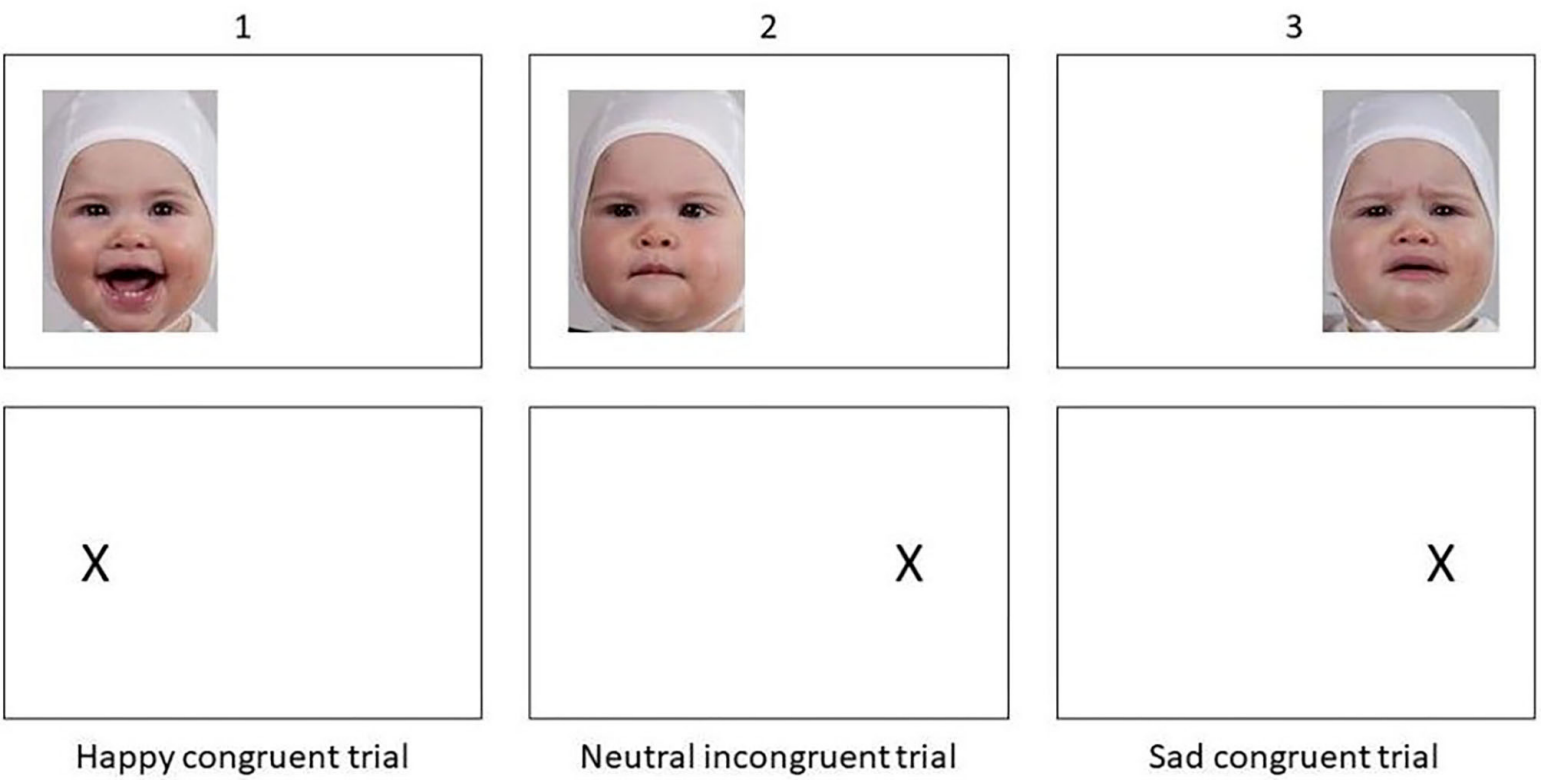
Example of trials from the dot-probe task.

To assess attentional bias, we adapted a well-known reaction time paradigm, a variation of the dot-probe task (MacLeod et al., 1986), where happy, sad and neutral infant faces served as stimuli. Each task trial started with a display of a fixation cross in the middle of the screen for 500 ms. A single stimulus image followed on either the left or the right side of the screen. The image was presented for 1,000 ms. Following the offset of an image, a dot-probe appeared on the same (congruent) or the opposite side (incongruent) of the screen. Participants pressed a key indicating the location of the dot-probe. The intertrial interval was 100 ms. A stimulus (a happy, sad or neutral infant face) appeared in the left or right position with equal probability (Figure 1). The task consisted of 10 rehearsal trials and then 144 trials (4 trials per image, 12 images per emotion). Before rehearsal trials, participants were presented with a written instruction for the task. Instructions told them that images of infant faces would appear, followed by an X on either the left or the right side of the screen. They were to focus on the image, and then press a key to indicate which side of the screen the X had appeared (E for left side, I for right side). After reading instructions, participants proceeded to the rehearsal trials and had the chance to consult with the test leader after rehearsal if they needed more instructions. The task was programmed using Inquisit (Millisecond software), which recorded response accuracy and latency to respond.

Previous studies have used stimulus-pairs where one image is of a neutral expression and the other of an emotional expression. Here, we presented only one image at a time, i.e., happy and sad facial expressions had no competing stimulus, and we included a neutral condition displaying an infant with a neutral facial expression. In this way, any bias that appears is not relative to the competing stimulus. Of note, as research on interpretation of both adult and infant faces has shown that depressed individuals interpret neutral faces more negatively (Stein et al., 2010; Gil et al., 2011; LeMoult and Gotlib, 2019), this version of the task allowed us to investigate biases to neutral facial expressions as well.

We assessed reliability by calculating the bias for each image and using McDonalds ω in Jasp (Trizano-Hermosilla and Alvarado, 2016; JASP, 2020). Reliability was acceptable for the neutral and sad condition, neutral: ω = 0.701, sad: ω = 0.669, but poor for happy: ω = 0.518. Reliability was higher in the MDD sample; neutral: ω = 0.718, sad: ω = 0.735, happy: ω = 0.566 than in the control group; neutral: ω = 0.709, sad: ω = 0.583, happy: ω = 0.482.

#### Face Recognition Task

To assess bias in recognition memory, we used a modified version of the Deese-Roediger-McDermott (DRM) paradigm (Deese, 1959; Roediger and McDermott, 1995). Participants were given written instructions to pay close attention to a series of faces and try to remember them. There were four series with adult faces and 10 series with infant faces. A series contained 12 (adult) or 10 (infant) sample images and five test stimuli. For the adult series the sample images were six persons, three males, three females, showing two expressions. The emotional expressions used in the adult series were anger, disgust, happiness, sadness, surprise, and neutral. The adult images were taken from the KDEF (Lundqvist et al., 1998). Pilot experiments showed that the adult faces are easier to discriminate. Therefore, in the infant series each series consisted of five infants showing two different facial expressions. The emotional expressions in the different series were fear, disgust, sadness, surprise, happiness and neutral. Only two emotions were displayed in each series. Images were presented long enough for elaborative associations (2,000 ms), but participants were not asked to appraise the images, as in the studies with more explicit processing of the emotional elements (Gilboa-Schechtman et al., 2002; Ridout et al., 2003). Rather, they were simply told to remember the images. The instructions thereby neither directed their attention to nor away from the emotional elements in the image.

**Figure 2:**
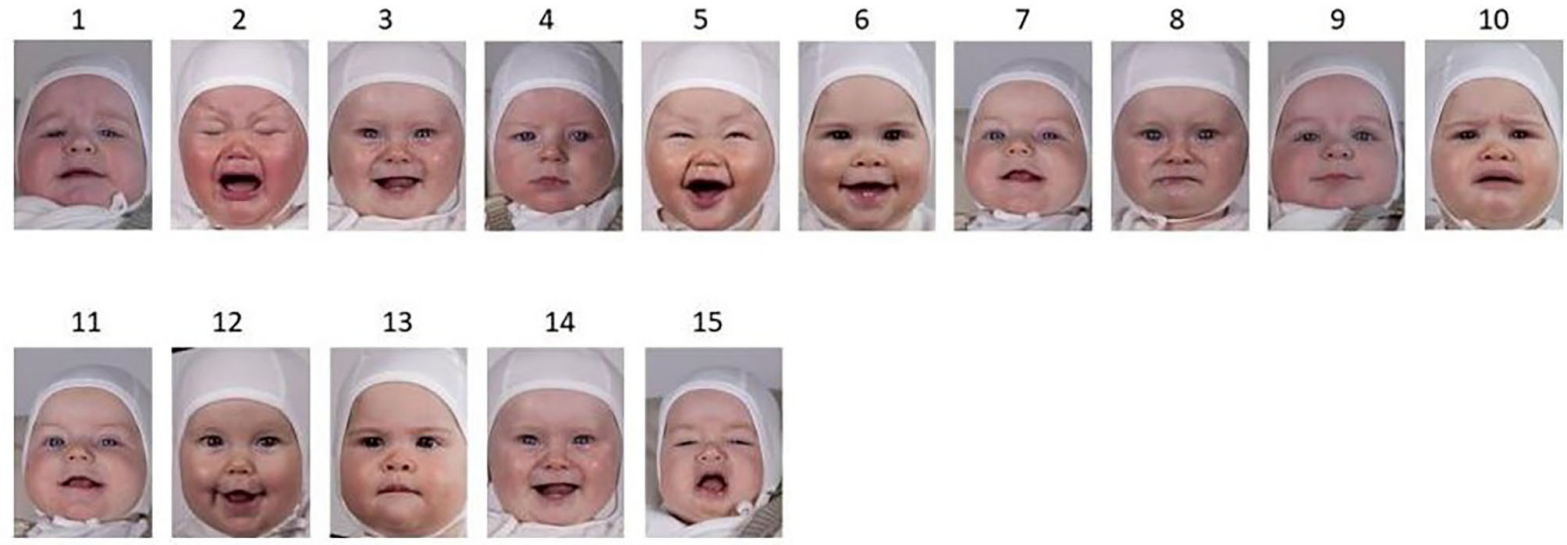
Example of a series from the Face Recognition Task. Top line: stimulus images, positive (happy) and negative (sad) valence. Bottom line: presented images for recognition. 11 = 7 (hit), 12 = face not seen, expression (happy) seen, 13 = face seen, expression (neutral) not seen, 14 = 3 (hit), 15 = face not seen, expression (angry) not seen.

Following each series of images, five further images were presented, and participants had to indicate if these images were in the series they had just seen. Answer options were “Definitely seen,” “Probably seen,” “Probably not seen,” and “Definitely not seen.” Two of the presented pictures had been in the series, whereas three of them had not. Of the non-presented images, one of them was of a seen face, but expressing a not-seen expression. One was of a face not seen, but with a seen expression. The last was of a face not seen with a not-seen expression (Figure 2).

#### Self-Report Measures of Depression and Rumination

All participants completed the Becks Depression Inventory-II (BDI-II; Beck et al., 1996) and the Ruminative Response Scale (RRS) from the Response Style Questionnaire (Nolen-Hoeksema, 1991; see Table 1 for group means). BDI-II is a widely used self-report questionnaire for measuring severity of depression. It has 21 items scored from 0 to 3, and a range of 0–63 where scores from 14 to 19 indicates mild depression, 20 to 28 moderate depression, and >28 severe depression (Beck et al., 1996). The RRS is a questionnaire that assesses participants tendency to ruminate when in a negative mood. It has 22 items, scored from 1 to 4, giving a range of 22–88. Higher scores indicate higher tendency to ruminate. Reliability of the BDI-II was ω = 0.942 and of the RRS was ω = 0.953.

#### Procedure

Participants completed the tasks and questionnaires on a computer, OS Windows 7, with a 23-inch screen. To ensure a standardized environment, participants in both the patient group and the control group completed the task in a computer lab, at the hospital or university, respectively. Prior to their completion, participants were informed about the project and gave their informed consent. Participants first completed the dot-probe task, and then answered demographic questions and the two questionnaires. They finished with the face recognition task where they first took the four series of adult faces and then the 10 series with infant faces. It took ~35 min to complete the questionnaires, the dot-probe task and the recognition task. At all times, a test leader was present, so participants could ask questions. When finished, participants were thanked for their contribution, and the control group received a gift certificate of 150 NOK (~$15), to compensate travel to the university.

#### Ethics

The Data Protection Official for Research approved data collection from the healthy group (NSD project no 45676), whilst the Regional Committee for Medicine and Health Research Ethics (REK project no 2014/2355) approved data collection from the depressed group.

### Results Study 1

#### Participant Characteristics

Demographics and clinical characteristics are presented in Table 1. Matching of the groups ensured that they did not differ according to age or parenthood. A chi-square test yielded no difference between the patient group and the control group in education level, χ^2^_(3)_ = 4.987, *p* = 0.173. As expected, there was a clear group difference in scores for BDI-II, *t*_(48)_ = 9.018, *p* < 0.001, *d* = 2.55, and RRS, *t*_(48)_ = 7.945, *p* < 0.001, *d* = 2.25.

#### Dot-Probe Task

Three participants had misunderstood the dot-probe task, leaving 23 from the control group and 24 from the patient group for analyses. The groups did not differ significantly on the demographic variables [education level χ^2^_(3)_ = 4.497, *p* = 0.106] but still differed in their BDI-II, *t*_(45)_ = 8.699, *p* < 0.001, *d* = 2.538 and RSS, *t*_(45)_ = 7.632, *p* < 0.001, *d* = 2.227. Only correct responses were analyzed. Error rates were very low (<1% in each group) and did not differ between the groups. Reaction times <200 ms or >950 ms were also excluded as these likely reflects lapses of concentration (cf. Gotlib et al., 2004). Average reaction time for each condition was then computed for each participant. The conditions were 3 (emotion: happy, sad, neutral) × 2 (congruent, incongruent). Attentional bias scores were computed separately for each emotion, using the following equation (Mogg et al., 1995):

$$\begin{array}{l} Attentional\;bias=0.5*\left(RT_{EI}-RT_{EC}\right) \end{array}$$

where RT is the mean response time, E = emotion, C = congruent, I = incongruent. This equation subtracts the mean probe detection times for congruent trials from the mean probe detection times for incongruent trials. A positive value indicates attention toward the stimulus image, whereas a negative value indicates disengagement from the stimulus image. As there was no competing stimulus in this version of the task, all values are negative because of the natural tendency to turn attention to the blank side of the screen.

**Figure 3:**
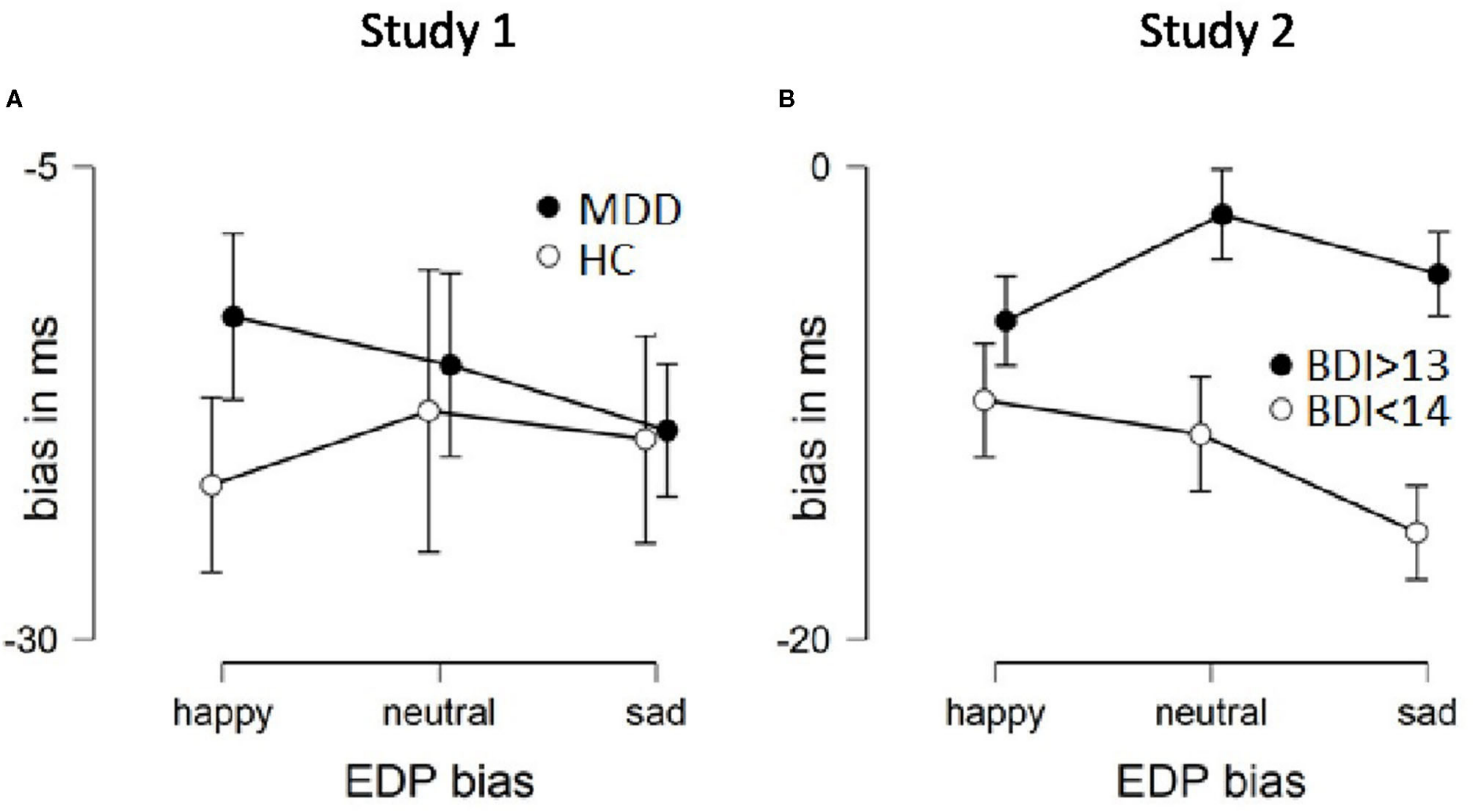
Attentional bias per group and emotion from both studies. Error bars are SEM. More negative values indicate faster disengagement.

Attentional bias for all three emotions and both groups were negative, i.e., responding was slower to congruent stimuli (average ranges from −13 ms to −22 ms). To examine group differences in attentional bias, we performed a mixed ANOVA (sphericity violation corrected with Greenhouse-Geisser) with emotion (happy, neutral, sad) as within factor and group as between factor. There was no difference for bias per emotion, *F*_(1.665, 74.488)_ = 0.122, *p* = 0.848, η^2^ = 0.003; nor was there a group difference, *F*_(1, 45)_ = 0.195, *p* = 0.661, η^2^ = 0.004. The interaction was not statistically significant, *F*_(1.665, 74.488)_ = 0.365, *p* = 0.655, η^2^ = 0.008 (Figure 3A). As the two groups did not differ, hypothesis 1 and 2 were refuted in this sample.

We also assessed whether the biases are significantly different from zero, per group, following Joormann and Gotlib (2007), i.e., using one sample *t*-tests (*p*-value corrected for multiple testing). Values close to zero indicates equal response time between congruent and incongruent trials. The control group had a significant attentional bias for sad [*t*_(22)_ = −2.931, *p* = 0.008, *d* = 0.611] and happy [*t*_(22)_ = −2.973, *p* = 0.007, *d* = 0.62] but not for neutral [*t*_(22)_ = −1.99, *p* = 0.059, *d* = 0.415]. Similarly, the MDD group had a significant attentional bias for sad [*t*_(23)_ = −2.89, *p* = 0.008, *d* = 0.59], but not for happy [*t*_(23)_ = −2.117, *p* = 0.045, *d* = 0.432] or neutral [*t*_(23)_ = −1.796, *p* = 0.086, *d* = 0.367]. Thus, both groups respond faster for sad incongruent trials than congruent trials, which indicate that they are faster at turning their attention away from the image in the sad condition. Only the control group shows an attentional bias for happy infant faces.

Since previous studies (Pearson et al., 2013) assessed the bias in pregnant women, or in mothers, we also controlled for having children. We did an exploratory mixed ANOVA with motherhood (yes or no), and age groups (seven age groups), did not yield a statistically significant effect either (see SOM, Table 1).

Trapp et al. (2018) recently reported no overall group difference but an effect for those with a pronounced depressive mood compared to the control group and the less depressive group. To explore this, we did a median split by BDI-II in the MDD group. Supplementary Figure 1 shows a similar pattern as Trapp et al. found, i.e., the more severe the current depressive mood as measured with the BDI-II, the more they engaged with the infant image regardless of valence, as seen in a bias closer to zero. However, this did not reach statistical significance (SOM, Table 2, Supplementary Figure 1).

**Table 2:** Participant characteristics study 2.

	**Depressive group**	**Control group**
	***M (SD)***	***M (SD)***
Age	29.6 (4.0)	31.2 (5.6)
BDI-II	18.9 (6.6)	5.5 (3.4)
RRS	40.7 (12.1)	32.7 (9.6)
	***N***	***N***
Female/male	21/8	21/8
Higher education^a^	19	19

a *Studied in university or college*.

Finally, we calculated person-centered percent correct classifications (PCC) effect sizes (Grice et al., 2020). Of the 23 matched pairs, in 13 pairs (57%) the depressed participant had a smaller bias than the matched control participant. This confirms the ANOVA results, i.e., the two groups are not easily distinguishable based on their bias in the emotional dot probe task.

#### Face Recognition Task

**Figure 4:**
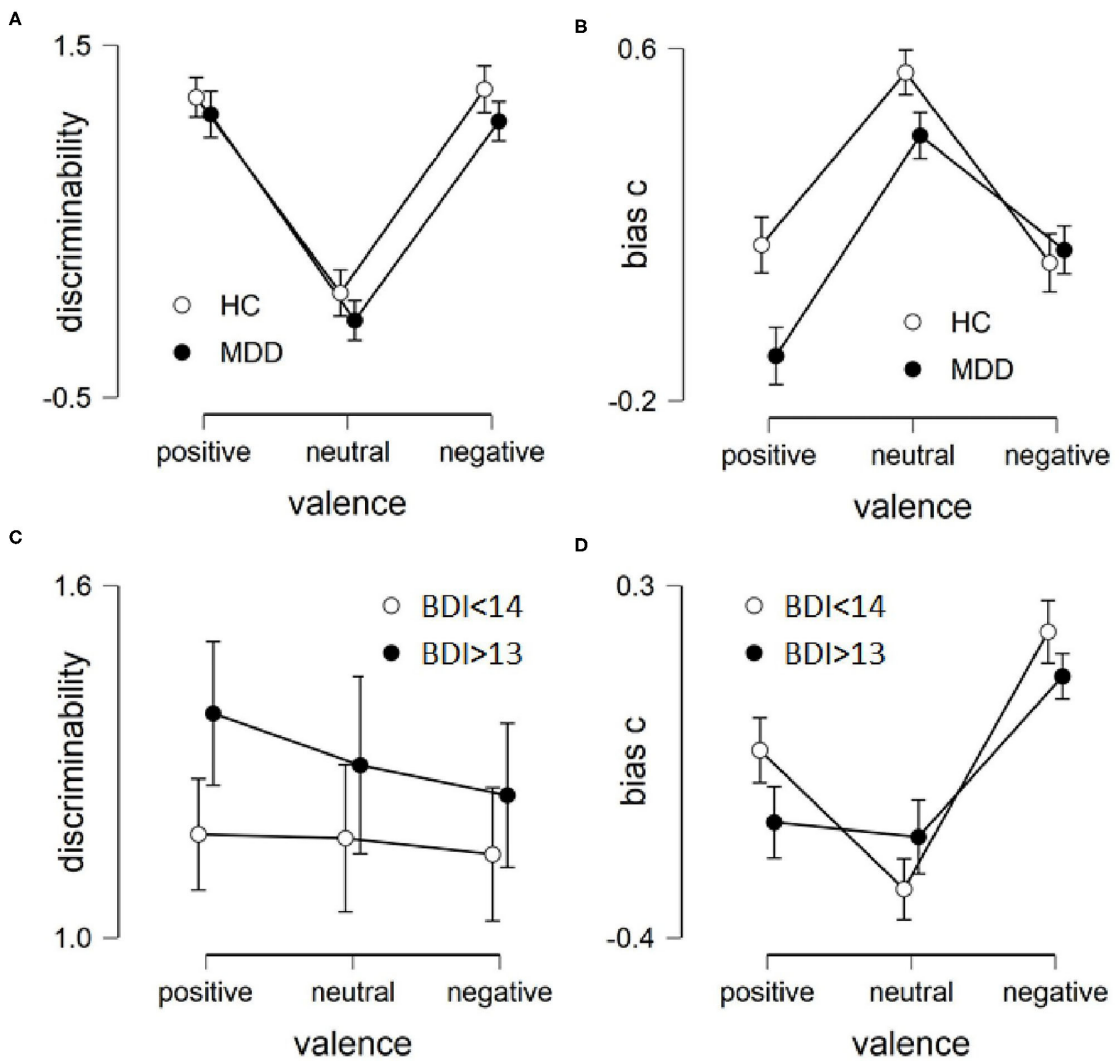
Results of recognition task per group and emotion from both studies. Left row is accuracy in recognition, higher values indicate higher accuracy. **(A)** Study 1, **(C)** Study 2. Right row is bias, the tendency to say yes to having seen the images. Higher values indicate more reserved in saying yes. **(B)** Study 1, **(D)** Study 2. Error bars are SEM.

All 50 participants completed this task. Participants *d'* and *bias c* were calculated according to signal detection theory (Macmillan and Creelman, 2004). Participant *d'* refers to their memory discriminability or accuracy, while *bias c* refers to a liberal or conservative tendency to judge an item as seen. A liberal bias, *c* < 0, is also referred to as “yes-sayer,” whereas a conservative bias, *c* > 0, results from more misses than false alarms. For adult faces, a *t-*test for independent samples revealed no significant group difference in general recognition accuracy (*d'*), *t*_(48)_ = 0.899, *p* = 0.373, *d* = 0.255. For infant faces, a *t-*test for independent samples revealed no significant group difference in general recognition accuracy (*d'*), *t*_(48)_ = 0.738, *p* = 0.464, *d* = 0.209. To examine for mood-congruent memory retrieval for infant faces, *d*' was calculated for three valence conditions; negative (sad, afraid, disgusted), positive (happy, surprised) and neutral. A mixed ANOVA revealed a significant effect of valence, *F*_(2, 96)_ = 58.432, *p* < 0.001, η^2^ = 0.411, but there was no interaction effect by group *F*_(2, 96)_ = 0.064, *p* = 0.938, η^2^ = 0; nor was there a main effect of group, *F*_(1, 48)_ = 1.458, *p* = 0.233, η^2^ = 0.029. The effect of valence is due to both groups being less accurate in recognizing neutral faces (Figure 4A). Although there were fewer rounds of adult faces, we compared negative facial expressions with positive and neutral expressions for adult stimuli as well. A mood congruent bias was not found for adult faces, i.e., neither was there a difference by valance, *F*_(1, 48)_ = 1.121, *p* = 0.295, η^2^= 0.007, by group, *F*_(1, 48)_ = 0.06, *p* = 0.808, η^2^= 0.001, or an interaction, *F*_(1, 48)_ = 0.614, *p* = 0.437, η^2^ = 0.004.

There was no group difference in participants' bias c for adult faces, *t*_(48)_ = 0.055, *p* = 0.957, *d* = 0.015, or infant faces, *t*_(48)_ = 1.2, *p* = 0.236, *d* = 0.34. To examine for mood-congruent memory bias, participant bias (*c*) was calculated for each valence condition for the infant faces. A repeated measures ANOVA revealed a significant effect of valence, *F*_(2, 96)_ = 31.531, *p* < 0.001, η^2^ = 0.215, no interaction effect by group *F*_(2, 96)_ = 2.884, *p* = 0.061, η^2^ = 0.02; and no main effect of group, *F*(1, 48) = 2.514, *p* = 0.119, η^2^ = 0.05. The effect of valence is due to both groups being more conservative toward neutral images (Figure 4B). For adult faces we found a non-significant difference in bias c by group, *F*_(1, 48)_ = 3.877, *p* = 0.055, η^2^ = 0.075, i.e., the MDD group was more liberal toward positive and neutral facial expressions. There was no main effect of emotion, *F*_(1, 48)_ = 0.801, *p* = 0.375, η^2^= 0.011, or an interaction, *F*_(1, 48)_ = 0.844, *p* = 0.363, η^2^ = 0.011. Overall, hypothesis 3 and 4 were refuted.

#### Rumination and Depression Severity

All 50 participants answered both questionnaires. In line with hypothesis 5, there was a strong correlation between the BDI-II score and the RSS score, Pearson's *r*_(48)_ = 0.796, *p* < 0.001; and the patient group had higher scores (Table 1). Contradictory to our hypothesis 6, rumination was not related to attentional bias in any valence condition, all *r*_(45)_'*s* < 0.123, *p's* > 0.4. The face recognition task was also not related to rumination, neither in discriminability [*r*_(48)_'s −0.201 to 0.05, *p'*s > 0.14] nor recognition bias [all *r*_(48)_'s > −0.19 and <0.11, *p'*s > 0.16]. No correlation was observed between the BDI-II scores and the attention biases [all *r*_(45)_'s < 0.1, *p*'s > 0.49] and recognition accuracies [*r*_(48)_'s > −0.11, *p*'s > 0.41] or biases [*r*_(48)_'s < 0.08, *p'*s > 0.29].

### Discussion Study 1

In study 1, we investigated cognitive biases in attention and recognition memory for infant facial expression in a clinically depressed group, women with MDD. We found neither attentional nor memory biases in the depressed group, compared to healthy controls. There was a strong correlation between depression severity measured by the BDI-II and the tendency to ruminate measured by the RSS, and the groups clearly differed from each other on these two measures. However, there were no significant correlations between rumination and the cognitive tasks. Except for the correlation between BDI-II and RRS, all our hypotheses were refuted.

In this study, we applied a modified version of the dot-probe task, which complicates the comparison with previous studies on adult faces. The present results are inconsistent with studies on attentional bias to adult faces using the traditional dot-probe task. This inconsistency could be caused by the modified version of the task. If that is the case, it would indicate that the established attentional biases do not occur when there are no competing stimuli, as in our version of the task. Notably, our modified dot-probe task had higher reliabilities than reported for the traditional task (Schmukle, 2005; Price et al., 2015).

Regardless, mood-congruent biases in depressed individuals are well-established, in different forms of cognitions and methodology (LeMoult and Gotlib, 2019). Results may therefore also be explained by the stimuli being infant faces. Perhaps the baby schema may protect against biased attention, or infant faces might not be salient enough to trigger bias in the patient group.

Regarding recognition memory, there were no group differences in accuracy to either infant or adult faces. Both the control and the patient groups were less accurate in recognizing neutral infant faces than happy or sad infant faces. Emotional infant facial expressions seem to take precedence over neutral expressions in memory, in both groups. Both groups' bias in recognition also reflected this, where participants were less likely to say yes to having seen neutral infant faces. Regarding adult faces, there was no effect of valence or group in either accuracy or bias, except for a non-significant trend in the depressed individuals to be more conservative toward negative adult faces. Ridout et al. (2009) also failed to demonstrate mood-congruent recognition of adult faces when comparing a depressed and non-depressed group. However, they did demonstrate such mood-congruent bias in another study, where they had participants identify the emotion of the face at encoding (Ridout et al., 2003). So it seems explicit processing of the emotional expression might be necessary to provoke mood-congruent bias in recognition memory.

Since the MDD-group in study 1 did not include parents of young children, this might have influenced the results. Previous research suggests that depression-related biases are more commonly observed when processing self-relevant material (Howe and Malone, 2011; Koster et al., 2011; LeMoult and Gotlib, 2019). Infant faces would not be of high relevance to the participants of study 1, as only one woman in the patient group had children under the age of 3. Therefore, we decided to investigate cognitive biases in a group of expecting parents reporting depressive symptoms.

## Study 2: Biases in Prenatal Depression

According to a systematic review by De Carli et al. (2019), pregnancy might affect facial processing. This is thought to be an evolutionary function, where increased vigilance in social appraisal might be essential in averting potentially harmful situations and assuring offspring survival. Facial processing is also essential to perceive and understand an infant's cues, which is necessary to provide sensitive care. Therefore, increased orientation toward facial expressions during pregnancy could favor both fetal protection and sensitive parenting. However, it is unclear in what way facial processing during pregnancy is affected. As De Carli et al. (2019) points out, there seems to be a difference in how emotional faces are perceived during pregnancy, but studies do not agree on which specific emotions elicit which specific responses.

While facial processing during pregnancy in general seems to be affected, the present study is specifically interested in the influence of depressive symptoms during pregnancy. As previously mentioned, studies by Pearson et al. (2010, 2013) indicate that depressed pregnant women are less engaged in infant distress than non-depressed pregnant women. However, stimulus presentations in these studies were only 240 ms, and according to previous research on attentional biases to adult faces, one would not expect a mood-congruent bias in the depressed group under these conditions. Looking at EEG-responses to infant faces, Rutherford et al. (2016) found a reduced response in pregnant women with higher depression scores, when presented with distressed infant faces for 500 ms. Again, this presentation time is too short to examine a mood-congruent bias (Mathews and MacLeod, 2005). Further, Rutherford et al. (2016) found no difference in EEG-responses to happy or neutral infant faces, and also no differences between the groups to auditory stimuli of infants crying (2,000 ms). In another study, Pearson et al. (2012), measured pulse and blood pressure while participants watched distressed infant faces for 6 s, and found increased systolic blood pressure in the depressed group compared to controls. Murphy et al. (2015) found higher levels of cortisol in the saliva of pregnant women with elevated depressive symptoms compared to non-depressed controls, after they watched a film of infants crying. Contradictory to the studies on attentional bias (Pearson et al., 2010, 2013), these studies indicate that the depressed group have higher sympathetic activation when faced with distressed infants. It is unclear why this occurs as activation could indicate both an approach and an avoidance response. Macrae et al. (2015) let pregnant women look at pictures of happy, neutral, and distressed infant faces, and had them rate how much they would like to comfort and how much they would like to turn away. They found that women with depression were more likely to turn away from any emotion, and less likely to comfort distressed infant faces, which indicates an avoidance response. However, Murphy et al. (2015) found no group difference in desire to comfort the infants after their participants had watched the film of crying infants.

Importantly, there might be a difference between interpretation and attention. In a review, De Carli et al. (2019) reported a possible mood-congruent interpretation of infant faces in depressed individuals, where they perceived distressed and neutral infants as sadder than healthy controls. This is in line with research on processing of adult faces. It seems an interpretation bias is more consistent than attention and recognition bias.

Pearson et al. (2011) found that healthy pregnant women's attention to distressed infant faces predicted their bonding to the infant after birth. Women who were less attentive to infant distress reported poorer bonding to the infant 3–6 months after birth. Depressive symptoms were not related to bonding. In a recent study, Dudek and Haley (2020) found that a stronger neural response during pregnancy to distressed infant faces vs. non-distressed faces was related to maternal sensitivity after birth. However, depressive symptoms were neither related to sensitivity nor attentional bias in this study. Note that both studies used a stimulus presentation time of only 240 ms, which can detect only initial engagement with the stimulus and not prolonged engagement or disengagement. Regardless, these studies show how initial engagement with the infant's negative emotions is an adaptive behavior that ensures sensitive care, and that preferential processing of these cues are detectable prenatally. Initial engagement with negative infant faces seems to be adaptive, but maybe not related to depression.

In summary, it is still unclear in what way prenatal depression affects infant face processing, as results are inconsistent. Some studies seem to replicate mood-congruent findings from the literature on adult faces, while other studies find indications of the opposite, namely avoidance of infant distress in depression. Others find signs of increased activation when presented with infant distress, but we cannot know if the activation is a sign of engagement or avoidance. Further, there are methodological issues with respect to stimulus presentation time that complicate reaching conclusions.

To shed more light on how prenatal depression affects processing of infant facial expressions, we studied a sample of expecting parents. Our hypotheses were the same as in study 1, where we expected mood-congruent biases to appear, in line with research on processing of adult faces in depression. As in study 1, we expected biases to be related to rumination and depressive symptoms.

### Materials and Methods

#### Participants

The sample for this study was extracted from a larger study; the Northern babies longitudinal study (NorBaby; for details see Høifødt et al., 2017). In NorBaby, pregnant women and their partners were recruited through midwife-services and followed throughout the pregnancy and the postnatal period until the child was ~7 months old. Altogether there were six assessment points, three during pregnancy and three after birth. For the present study, a group consisting of 29 participants with BDI-scores above cut-off for mild depression (>13; Beck et al., 1996) was matched with a group of 29 participants with BDI-scores below the cut-off (see Table 2 for participant characteristics).

#### Materials

In the NorBaby study, participants answered several questionnaires, but for the present study we extracted only relevant questionnaires and test results, namely the same as in study 1; BDI-II, RRS, the emotional dot-probe task and the face recognition task, in addition to demographic information. As NorBaby is a longitudinal study with six points of assessment (T1 to T6), the face recognition task and the RRS were completed at later time points (T2 and T3, respectively) than the BDI-II and EDP (T1), although all of them took place during the last two trimesters of pregnancy. The materials and tasks were the same as in study 1. Reliability of the BDI-II was ω = 0.882 and of the RRS it was ω = 0.933.

Reliability of the EDP-task was calculated as in study 1, for each image and using McDonalds ω in Jasp (Trizano-Hermosilla and Alvarado, 2016; JASP, 2020). Reliability scores were for: neutral images ω = 0.548, sad images ω = 0.483, happy images ω = 0.465.

#### Procedure

Participants in the NorBaby study met a member of the research group after agreeing to participate via phone and answered T1 with the research group member present. T2 and T3 were sent to participants via e-mail, and participants answered at home in their own time.

#### Analysis

We used propensity scoring (Ho et al., 2011) (gender, number of children, and education) to match the depressed sample to a control group. EDP bias and recognition memory were analyzed as in study 1.

### Results Study 2

#### Participant Characteristics

Among 352 participants from the NorBaby study there were 29 participants (21 women, 8 men, age range 17 to 41) with valid EDP data who had a BDI-II score equal or larger than 14. We refer to those 29 participants as the depressed group. A *t*-test showed there was no difference between the depressed group and the propensity matched control group in age, *t*_(56)_ = 1.22, *p* = 0.227, *d* = 0.32. As expected, there was a clear group difference in scores for BDI-II, *t*_(56)_ = 9.747, *p* < 0.001, *d* = 2.56, and RRS, *t*_(41)_ = 2.389, *p* = 0.022, *d* = 0.73.

#### EDP Bias

Attentional bias for all three emotions and both groups were negative, i.e., responding was slower to congruent stimuli (average ranges from −2 ms to −15 ms). To examine group differences in attentional bias, we performed a mixed ANOVA with emotion (happy, neutral, and sad) as within factor and group as between factor. There was no difference for bias per emotion, *F*_(2, 112)_ = 1.289, *p* = 0.28, η^2^ = 0.008; but a group difference, *F*_(1, 56)_ = 5.881, *p* = 0.019, η^2^ = 0.095. The interaction was not statistically significant, *F*(_2, 112)_ = 1.834, *p* = 0.165, η^2^ = 0.011 (Figure 3B). Following Joormann and Gotlib (2007) one-sample *t*-tests were conducted comparing attentional bias scores to zero within each group. A value close to zero means that reaction time is similar between congruent and incongruent trials. The depressed group's biases were not significantly different than zero for sad [*t*_(28)_ = −1.698, *p* = 0.101, *d* = 0.31] and neutral [*t*_(28)_ = −0.734, *p* = 0.469, *d* = 0.136] faces, but for happy [*t*_(28)_ = −2.977, *p* = 0.006, *d* = 0.553]. In contrast, the matched control group disengaged for all three emotions faster from the infant faces in incongruent trials than congruent trials, i.e., sad [*t*_(28)_ = 5.381, *p* < 0.001, *d* = 1], neutral [*t*_(28)_ = 3.4, *p* = 0.002, *d* = 0.631], and happy [*t*_(28)_ = 3.115, *p* = < 0.004, *d* = 0.578]. Together with the group difference from the ANOVA these results partly confirm our hypothesis 2. The depressed group did not disengage as easily from sad faces as did the control group [independent *t*-test of sad faces confirms this: *t*_(56)_ = 2.784, *p* = 0.007, *d* = 0.731]. Hypothesis 1 is refuted as both groups are slower to disengage from congruent happy faces [independent *t*-test finds no group difference to happy faces, *t*_(56)_ = 0.871, *p* = 0.388, *d* = 0.229].

We next calculated the person-centered PCC (Grice et al., 2020). The attentional bias for sad was 69%, i.e., in more than 2/3 of the cases group membership would be correctly classified. For happy and neutral the PCC was 62%. Overall, the PCC was 64%.

#### Face Recognition Memory

There was some attrition from T1 to T2 yielding only *n* = 25 in the depressed group. Still, the groups differed according to their BDI-II-scores [*t*_(48)_ = 8.82, *p* < 0.001, *d* = 2.49] and RRS-scores [*t*_(39)_ = 2.4, *p* = 0.021, *d* = 0.75]. There was no group difference in overall discriminability for adult faces [*t*_(48)_ = 0.721, *p* = 0.475, *d* = 0.204], or infant faces [*t*_(48)_ = 1.053, *p* = 0.298, *d* = 0.298]. A mixed ANOVA revealed no significant effect of valence for infant faces, *F*_(2, 96)_ = 0.251, *p* = 0.779, η^2^ = 0.003, no interaction effect by group *F*_(2, 96)_ = 0.101, *p* = 0.904, η^2^ = 0.001; and no main effect of group, *F*_(1, 48)_ = 1.097, *p* = 0.3, η^2^ = 0.022 (Figure 4C). For adult faces, comparing negative with positive and neutral facial expressions, there was a main effect for valence, *F*_(1, 49)_ = 4.298, *p* = 0.043, η^2^ = 0.041, but no difference by group, *F*_(1, 49)_ = 0.408, *p* = 0.526, η^2^ = 0.008, or an interaction, *F*_(1, 49)_ = 0.148, *p* = 0.702, η^2^ = 0.001. Discriminability of adult faces was higher for positive and neutral expressions.

Regarding bias in recognition memory, there was no group difference for overall bias for adult faces [*t*_(48)_ = −0.373, *p* = 0.711, *d* = −0.106], or infant faces [*t*_(48)_ = 0.62, *p* = 0.538, *d* = 0.175]. A repeated measures ANOVA revealed a significant effect of valence for infant faces, *F*_(2, 96)_ = 21.943, *p* < 0.001, η^2^ = 0.181, no interaction effect by group *F*_(2, 96)_ = 2.091, *p* = 0.129, η^2^ = 0.017; and no main effect of group, *F*_(1, 48)_ = 0.336, *p* = 0.565, η^2^ = 0.007. The effect of valence is due to both groups being more conservative toward negative images (Figure 4D). For adult faces, we found a main effect of valance, *F*_(1, 49)_ = 8.6, *p* = 0.005, η^2^ = 0.082, i.e., both groups were more liberal toward positive and neutral facial expressions than negative adult faces. There was no main effect of group, *F*_(1, 49)_ = 0, *p* = 0.998, η^2^ = 0, or an interaction, *F*_(1, 49)_ = 0.671, *p* = 0.417, η^2^ = 0.006.

Overall, we found no support for hypothesis 3 or 4.

#### Rumination and Depression Severity

As in study 1, there was a strong correlation between the BDI-II score and the RSS score, Pearson's *r*_(46)_ = 0.57, *p* < 0.001; and the depressed group had higher scores (Table 2).

In line with our hypothesis 6, rumination was, correcting for multiple testing, positively related to attentional bias for neutral and negative (sad) images, *r_neutral(46)* = 0.368, *p* = 0.015, *r_sad(46)* = 0.372, *p* = 0.014 but not for happy images, *r_happy(46)* = 0.222, *p* = 0.153. Regarding the recognition task we found no relationship between rumination and discriminability, all *r*'s < 0.13, *p's* > 0.42, or bias, all *r*‘s < 0.2, *p's* > 0.19.

Also in line with our hypothesis 6, more severe depressive symptoms were positively related to attentional bias for neutral and negative (sad) images, *r_neutral(58)* = 0.284, *p* = 0.031, *r_sad(58)* = 0.353, *p* = 0.007 but not for happy images, *r_happy(58)* = 0.079, *p* = 0.557. Similarly to rumination, depressive symptoms had no relationship to discriminability, all *r's* < 0.19, *p's* > 0.18 and bias, all *r's* < 0.17, *p's* > 0.24 in the recognition memory task.

#### Comparing Study 1 and 2

As there were group differences in study 2 but not in study 1, we checked if the two studies were significantly different regarding attentional bias. Since we had a small sample size in both studies, we consider both *p*-value and effect size. For attentional bias we found that the difference between the MDD group (study 1) and the depressed group (study 2) had a medium effect size, *F*_(1, 51)_ = 3.227, *p* = 0.078, η^2^ = 0.06.

In contrast to study 1, in study 2 there was a significant correlation between the attentional bias to sad faces and both rumination and depression severity. However, the correlation between depression severity and bias to sad faces in study 1 [*r*_(48)_ = 0.096] was not significantly different from study 2 [*r*_(58)_ = 0.353; *z* = 0.929, *p* = 0.176], neither was the correlation between rumination and bias to sad faces in study 1 [*r*_(48)_ = 0.115] and study 2 [*r*_(58)_ = 0.372; *z* = 0.938, *p* = 0.174].

### Discussion Study 2

Testing expecting parents we found that the non-depressed group disengaged faster than the depressed group from sad faces, and there was a trend in the same direction with neutral faces. According to *post-hoc* testing, the group difference in attention to sad faces was particularly pronounced. This is in line with previous research on processing of adult faces where depressed individuals display a mood-congruent bias. It can also be understood as a lack of a protective bias, where the healthy group disengages from negative material faster, as this is adaptive in mood regulation. Comparing the two groups on attentional bias to neutral faces showed a trend in the same direction as with the sad faces, where the healthy group disengaged faster. This makes sense as research on interpretation of facial expressions of both adults and infants has shown that depressed individuals interpret neutral faces as more negative than healthy controls. There was no group difference in attention to happy faces. Regardless, our results from the groups of expecting parents are similar to previous research on adult faces and words (Joormann and Gotlib, 2007; Fritzsche et al., 2009; Grafton et al., 2016). Attentional bias for neutral and sad images was also related to rumination and depression. Slower disengagement from these images was related to higher levels of rumination and more depressive symptoms. This supports the impaired disengagement hypothesis, where one sees both rumination and mood-congruent attentional bias as a result of lower attentional control in the presence of self-relevant negative information (Koster et al., 2011). Also, this supports Joormann's model of cognitive inhibition and emotion regulation (2010).

Results on our attentional task adds knowledge to the findings of Pearson et al. (2010, 2013). These studies found an early orientation toward distressed infant faces in the non-depressed group, but not in the depressed. Such fast orientation toward infant cues would be an important part of sensitive parenting. On the other hand, maintained attention on distress in a baby who is not yours, and not yours to comfort, might not be adaptive as it would only serve to affect your mood. As Pearson's studies used a short stimulus presentation time, their task would not be sensitive to maintained attention. It is this maintained attention or lack of disengagement from negative material that characterizes depression, as is shown in both eye-tracking studies (Suslow et al., 2020) and reaction time studies (Mathews and MacLeod, 2005). Interestingly, both Dudek and Haley (2020) who found an association between maternal sensitivity and neural response to infant distress, and Pearson et al. (2011) failed to find association between depressive symptoms and maternal sensitivity or bonding, respectively. Thereby, initial attention allocation toward infant distress absolutely seems adaptive when preparing for parenthood, but it might not be a major feature of depression. Further, this highlights how depression is only a risk factor for less sensitive parenting, and that many depressed mothers are still capable of sensitive care for their infant. When examining biases characteristic of depression, we recommend a stimulus presentation time of at least 1,000 ms, as is demonstrated in other studies (Mathews and MacLeod, 2005; Stein et al., 2010).

For recognition memory there were no group differences. This is in line with the findings of Ridout et al. (2009). Regarding bias in recognition, the tendency to say yes or no to the images, both groups were more conservative toward negative faces. Notably, this bias differed from results in study 1. While in study 1 both groups were more conservative, saying less yes, to having seen neutral faces, this was not the case in study 2. In study 2, both groups were more conservative to negative faces. This is a peculiar finding. Being conservative toward neutral faces, as in study 1, might be caused by neutral faces being more easily forgotten as they are less salient stimuli. This is reflected in the accuracy, which is lower for neutral faces in both groups in study 1. In expecting parents, however, the accuracy of negative faces is not worse than that of positive and neutral faces, it is only the bias that is more conservative. It seems that the expecting parents do not overgeneralize infant distress. They have fewer “false alarms” to negative faces. This might be adaptive, as one does not create false memories of negative emotions. As this differs from study 1, it might be a characteristic of the pregnant or expecting mind.

## General Discussion

Altogether, the present two studies add important findings to our understanding of cognitive biases in depression. They indicate that material needs to be self-relevant for such biases to appear, as there were no group differences in study 1 where the participants were inpatients, a majority above reproductive age and only one woman had children of toddler-age. Infant faces were thereby not relevant for this group in their present life situation. In the group of expecting parents on the other hand, we observed both a mood-congruent attentional bias and a significant correlation between bias to sad and neutral faces, and rumination and depression severity. This is especially interesting as the group in study 2 was not diagnosed with depression, and both their rumination score and depression severity score were lower than the depressed group in study 1. This emphasizes the importance of self-relevant material in studies examining mood-congruent biases in depression. However, when comparing results of the two studies they were not statistically significantly different, and therefore one must be careful to conclude even though the trend was consistent.

Attentional bias results might be affected by our variant of the dot-probe task. Our version operates with only one stimulus at a time, instead of stimulus pairs. This is, however, more similar to the task used by Pearson et al. (2010) and Dudek and Haley (2020). When presented with stimulus pairs, a happy face would be paired up with a neutral face, and so a preference for the happy face would be relative to the neutral. Therefore, the positivity bias found in healthy controls processing adult faces (Joormann and Gotlib, 2007; Fritzsche et al., 2009), might reflect either a preference for happy faces over neutral ones, or that depressed groups get caught up in the neutral image and not that they avoid the happy. One might expect effects to be more pronounced in the traditional task, where attentional bias reflects some sort of preference for one over the other image. However, one does not know if a group difference in processing of a stimulus pair with a happy and a neutral face would be caused by a preference for the happy face in the control group, or a preference for the neutral image in the depressed group. Therefore, our task can capture bias in a more specific way, not relative to other stimuli. In addition, our version lets us compare the groups' processing of neutral faces as well, which is not possible in the traditional version. Further, this version of the task had higher reliability than the standard version of the task (Schmukle, 2005; Staugaard, 2009; Waechter et al., 2014; Price et al., 2015). Future research should investigate this further by testing both versions of the dot-probe task on the same group.

Regarding recognition, there were no group differences in either study. The lack of group differences might be explained by the level of processing. The studies conducted by Ridout et al. (2003, 2009) suggest a difference between explicit and implicit recognition tasks. Only such explicit tasks, like directing participants to appraise the emotional features in a face, seem to be sufficient for provoking a mood-congruent bias. In that regard, we might have seen an effect of depression if the instructions had been to remember the emotional expressions instead of just the image.

### Limitations

The inclusion criteria for the patient group in study 1 was that they were admitted in the department for depressive disorders, meaning that their primary diagnosis was MDD. However, we did not control for secondary diagnoses, so some of the participants may have had comorbid anxiety or other disabilities. In terms of ecological validity though, individuals with MDD often experience complex symptoms, and MDD is heterogeneous (Goldberg, 2011). The patient group participated during a treatment stay, and so the ongoing treatment might have influenced results. Notably, most patients participated during the beginning of their stay, and their BDI-II scores confirmed high levels of depressive symptoms.

We believe our version of the dot-probe task is an improvement, as it has higher reliability and allows for examining bias to neutral faces. Even so, conclusions are limited as we cannot readily compare our results to previous research with the dot-probe task. Still, we see the results as complementary knowledge to what previous research has found, and hope that we can contribute to further development of methods and designs.

Regarding the face recognition task, the negative valence category included several emotional expressions rated as negative and not just sad expressions, which are mood-congruent with depression. This might affect the results. More research on the effect of depression on recognition memory of both adult and infant faces is needed.

We used images from the Tromsø infant face database (Maack et al., 2017), and did not use infant images from the parents' own child (e.g., Laurent and Ablow, 2013). It is possible that a mood-congruent bias would be present or more pronounced if we had used own-infant-images, as the participants then would have a relation to the infant. It would at least ensure self-relevance of the material. However, not all participants had children from before, and using participants own children would have complicated standardization of the tasks and images.

Our sample sizes were small, as it is difficult to recruit large clinical samples, and so we missed out on small group differences that can still be clinically relevant (Funder and Ozer, 2019). Looking at effect sizes, the difference between the studies are of medium size, supporting our interpretation of self-relevance.

## Conclusion

The present studies demonstrated that inpatient women with MDD do not display processing biases compared to a healthy control group when processing emotional infant faces in a reaction time attention task and a recognition memory task. At the same time, expecting parents with depressive symptoms differed from healthy controls in the same attention task, where the healthy group disengaged faster than the depressed group from sad infant faces. Attentional bias was also related to rumination in this sample. Results are supportive of the impaired disengagement hypothesis and highlight the role of self-relevant material for attentional bias to appear.

## Data Availability Statement

The datasets presented in this study can be found in online repositories. The names of the repository/repositories and accession number(s) can be found at: https://osf.io/zn5pr/?view_only=eab8dfed0db04f5e897d7a7df88251c3.

## Ethics Statement

In study 1, the Data Protection Official for Research approved data collection from the healthy group (NSD project no 45676), whilst the Regional Committee for Medicine and Health Research Ethics (REK project no 2014/2355) approved data collection from the depressed group. Study 2 was approved by the Regional Committees for Medical and Health Research Ethics, ref. 2015/614. The patients/participants provided their written informed consent to participate in this study.

## Author Contributions

AB, DN, ÅL, CW, and GP designed the study. GP developed the cognitive tasks. AB, DN, ÅL, and PU recruited participants and collected the data. GP analyzed the data. AB wrote the manuscript while all authors contributed in critical evaluation and editing of the manuscript. All authors contributed to the article and approved the submitted version.

## Conflict of Interest

The authors declare that the research was conducted in the absence of any commercial or financial relationships that could be construed as a potential conflict of interest.
