# Supplementary material for: Emotional Infant Face Processing in Women With Major Depression and Expecting Parents With Depressive Symptoms
Source: Front Psychol. 2021 Jul 2;12:657269. doi: 10.3389/fpsyg.2021.657269 (PMC8283203; doi:10.3389/fpsyg.2021.657269)

**Supplementary material**

# Supplementary table 1. Repeated Measures ANOVA for group, age and motherhood

| **Within Subjects Effects** | | | | | | | | | | | | | | | | | | | | | |
| --- | --- | --- | --- | --- | --- | --- | --- | --- | --- | --- | --- | --- | --- | --- | --- | --- | --- | --- | --- | --- | --- |
|  | | **Sphericity Correction** | | | **Sum of Squares** | | | **df** | | **Mean Square** | | | | **F** | | **p** | | **η²** | | **η² _p_** | |
| bias |  | Greenhouse-Geisser | |  | 3458.9 | | ᵃ | 1.601 | ᵃ | 2159.9 | | | ᵃ | 2.921 | ᵃ | 0.072 | ᵃ | 0.056 |  | 0.064 |  |
| bias ✻ group |  | Greenhouse-Geisser | |  | 441.4 | | ᵃ | 1.601 | ᵃ | 275.6 | | | ᵃ | 0.373 | ᵃ | 0.643 | ᵃ | 0.007 |  | 0.009 |  |
| bias ✻ age |  | Greenhouse-Geisser | |  | 2978.2 | | ᵃ | 1.601 | ᵃ | 1859.8 | | | ᵃ | 2.515 | ᵃ | 0.099 | ᵃ | 0.049 |  | 0.055 |  |
| bias ✻ motherhood |  | Greenhouse-Geisser | |  | 3449.4 | | ᵃ | 1.601 | ᵃ | 2154.0 | | | ᵃ | 2.913 | ᵃ | 0.072 | ᵃ | 0.056 |  | 0.063 |  |
| Residual |  | Greenhouse-Geisser | |  | 50919.4 | |  | 68.860 |  | 739.5 | | |  |  |  |  |  |  |  |  |  |
|  | | | | | | | | | | | | | | | | | | | | | |
| *Note.*  Type III Sum of Squares | | | | | | | | | | | | | | | | | | | | | |
| ᵃ Mauchly's test of sphericity indicates that the assumption of sphericity is violated (p < .05). | | | | | | | | | | | | | | | | | | | | | |
| **Between Subjects Effects** | | | | | | | | | | | | | | | | |  |  |  |  |  |
|  | | | **Sum of Squares** | | **df** | | **Mean Square** | | **F** | | **p** | | **η²** | | **η² _p_** | |  |  |  |  |  |
| group | |  | 435.1 |  | 1 |  | 435.1 |  | 0.163 |  | 0.689 |  | 0.003 |  | 0.004 |  |  |  |  |  |  |
| age | |  | 8855.8 |  | 1 |  | 8855.8 |  | 3.313 |  | 0.076 |  | 0.069 |  | 0.072 |  |  |  |  |  |  |
| motherhood | |  | 3199.1 |  | 1 |  | 3199.1 |  | 1.197 |  | 0.280 |  | 0.025 |  | 0.027 |  |  |  |  |  |  |
| Residual | |  | 114938.5 |  | 43 |  | 2673.0 |  |  |  |  |  |  |  |  |  |  |  |  |  |  |
|  | | | | | | | | | | | | | | | | |  |  |  |  |  |
| *Note.*  Type III Sum of Squares | | | | | | | | | | | | | | | | |  |  |  |  |  |

# Supplementary table 2. Repeated Measures ANOVA, similar to Trapp et al. (2018) median split of MDD group in high and low severity, based on BDI-II.

| **Within Subjects Effects** | | | | | | | | | | | | | | | |
| --- | --- | --- | --- | --- | --- | --- | --- | --- | --- | --- | --- | --- | --- | --- | --- |
|  | | **Sphericity Correction** | | **Sum of Squares** | | **df** | | **Mean Square** | | **F** | | **p** | | **η²** | |
| bias |  | Greenhouse-Geisser |  | 259.6 | ᵃ | 1.644 | ᵃ | 157.8 | ᵃ | 0.203 | ᵃ | 0.773 | ᵃ | 0.005 |  |
| bias ✻ subgroup |  | Greenhouse-Geisser |  | 825.9 | ᵃ | 3.289 | ᵃ | 251.1 | ᵃ | 0.324 | ᵃ | 0.826 | ᵃ | 0.014 |  |
| Residual |  | Greenhouse-Geisser |  | 56132.6 |  | 72.354 |  | 775.8 |  |  |  |  |  |  |  |
|  | | | | | | | | | | | | | | | |
| Note.  Type III Sum of Squares | | | | | | | | | | | | | | | |
| ᵃ Mauchly's test of sphericity indicates that the assumption of sphericity is violated (p < .05). | | | | | | | | | | | | | | | |

| **Between Subjects Effects** | | | | | | | | | | | | | |
| --- | --- | --- | --- | --- | --- | --- | --- | --- | --- | --- | --- | --- | --- |
|  | | **Sum of Squares** | | **df** | | **Mean Square** | | **F** | | **p** | | **η²** | |
| subgroup |  | 4710 |  | 2 |  | 2355 |  | 0.853 |  | 0.433 |  | 0.037 |  |
| Residual |  | 121480 |  | 44 |  | 2761 |  |  |  |  |  |  |  |
|  | | | | | | | | | | | | | |
| Note.  Type III Sum of Squares | | | | | | | | | | | | | |

| **Descriptives** | | | | | | | | | |
| --- | --- | --- | --- | --- | --- | --- | --- | --- | --- |
| **bias** | | **subgroup** | | **Mean** | | **SD** | | **N** | |
| happy |  | HC |  | -21.830 |  | 34.51 |  | 23 |  |
|  |  | high_MDD |  | -8.417 |  | 33.10 |  | 12 |  |
|  |  | low_MDD |  | -17.435 |  | 33.82 |  | 12 |  |
| neutral |  | HC |  | -17.906 |  | 44.02 |  | 23 |  |
|  |  | high_MDD |  | -7.000 |  | 40.85 |  | 12 |  |
|  |  | low_MDD |  | -23.946 |  | 35.90 |  | 12 |  |
| sad |  | HC |  | -19.391 |  | 31.60 |  | 23 |  |
|  |  | high_MDD |  | -9.096 |  | 36.16 |  | 12 |  |
|  |  | low_MDD |  | -28.768 |  | 37.24 |  | 12 |  |
|  | | | | | | | | | |

# Supplementary figure 1.

Attentional bias in healthy controls and subgroups of the MDD-group based on BDI-II scores.


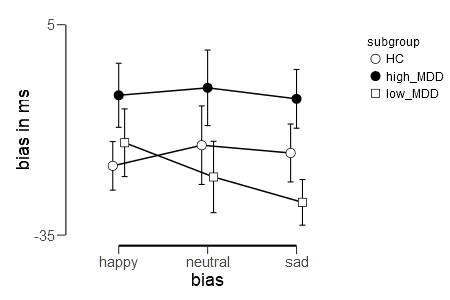

Supplement: Supplementary file 1 [file Data_Sheet_1.docx]
